# Low prevalence of *Neospora caninum* and *Toxoplasma gondii* antibodies in dogs in Jilin, Henan and Anhui Provinces of the People’s Republic of China

**DOI:** 10.1186/s12917-014-0295-3

**Published:** 2014-12-12

**Authors:** Yurong Yang, Qiongfang Zhang, Yangguang Kong, Yuqing Ying, Oliver Chun Hung Kwok, Hongde Liang, Jitender Prakash Dubey

**Affiliations:** Laboratory of Veterinary Pathology, College of Animal Science and Veterinary Medicine, Henan Agriculture University, Zhengzhou, 450002 PR China; United States Department of Agriculture, Agricultural Research Service, Beltsville Agricultural Research Center, Animal Parasitic Diseases Laboratory, Beltsville, MD 20705-2350 USA

**Keywords:** *Neospora caninum*, *Toxoplasma gondii*, Seroepidemiology, Dogs, China, Modified agglutination test, *Neospora* agglutination test

## Abstract

**Background:**

*Neospora caninum* and *Toxoplasma gondii* are important pathogens of worldwide distribution. *N. caninum* is a major cause of abortion in cattle and dogs are main reservoirs because they excrete the environmentally resistant oocysts. Toxoplasmosis is a worldwide zoonosis and dogs are considered as sentinels for this parasite because of their close contact with people and cats; additionally dog meat is also used for human consumption in China. The aim of the present study was to assess the prevalence of *N. caninum and T. gondii* infection in dogs from China. A total of 425 countryside dog hearts in Jilin, Henan and Anhui provinces of the People’s Republic of China were collected from slaughter houses in two batches; the first batch of 96 in October 2013, and the second batch of 329 in April 2014. Serum samples extracted from 96 dog hearts were tested for antibodies to *N. caninum* and from 425 dog hearts were tested for *T. gondii* antibodies in the modified agglutination tests (cut-off 1:25 for both), using respective antigens.

**Results:**

Antibodies to *N. caninum* were 6 of 96 (6.25%) of dogs with titers of 1:25 in 2, 1:50 in 3, and 1:100 in 1. All seropositive dogs were more than 1 year old. Antibodies to *T. gondii* were found in 35 of 425 (8.24%) dogs with titers of 1:25 in 15, 1:50 in 14; and 1:100 in 6.

**Conclusion:**

The results of the present study indicated low prevalence of *N. caninum* and *T. gondii* antibodies in dogs of China, compared with Europe and America. Identification of the risk factors that underlie these differences may help prevention of neosporosis and toxoplasmosis. This is the first report of *N. caninum* infection in dogs from China.

## Background

*Neospora caninum* and *Toxoplasma gondii* are related coccidians that until 1988 were considered the same organism [1]. *N. caninum* is now considered the most important abortifacient for cattle worldwide, including China [2,3]. Dog, wolf, coyote and dingo are the definitive hosts for *N. caninum* that shed environmentally resistant oocysts. *Toxoplasma gondii* infection in dogs is important for following reasons. Dogs can be infected through contact with the *T. gondii*, which may be acquired from rooting in infected soil or from ingesting cat feces or from eating raw meat. Dogs can also mechanically transmit *T. gondii* oocysts to humans [4]. In China, dogs serve as food animals, the consumption of undercooked meat containing *T. gondii* tissue cysts can be a supplementary health risk to consumers.

Currently, there is no report of isolation *T. gondii* from dog in China, and little is known of *N. caninum* infections in dogs in China. The objective of present study was to determine the seroprevalence of *N. caninum and T. gondii* infections in dogs from China, and to attempt isolate *T. gondii*.

## Methods

### Naturally infected dogs

A total of 425 countryside dogs were sampled from the slaughter house (Table 1). These countryside dogs in China are part of the farmer’s household; they were mainly used for guarding. Their diet includes boiled rice, discarded raw food animal tissues and whatever dogs can forage. These dogs were sold for food. Hearts of dogs were selected for the present study because serum could be obtained from the heart. The first batch samples (96 dog hearts) were collected in October 2013; 50 dogs were younger than 1 year, and 46 were older than 1 year. Second batch samples (329 dog hearts) were collected in April 2014; they were older than 1 year. Blood was collected from heart 1 day (second batch) or three days (first batch) after slaughter, centrifuged at 2000 × g for 10 min, and sera were separated. Hearts samples from first batch were transported by air as part of personal baggage of the senior author from China to the Animal Parasitic Diseases Laboratory (APDL), United States Department of Agriculture, Beltsville, Maryland, USA within one week of killing. During interim, samples were kept cold. Permission papers from USA and China were obtained before transportation.

**Table 1:** **Seroprevalence of** ***Toxoplasma gondii*** **and** ***Neospora caninum*** **infection in dogs in Jilin, Henan and Anhui Provinces**

**Characteristics**	**Dogs tested no.**	**Positive no. in different titres**				**Total positive no. (cut-off 1:25)**	**Seroprevalence (%)**	**95% CI**
		**1:10**	**1:25**	**1:50**	**1:100**			
**Toxoplasma gondii**								
**Region**								
**Henan**	31	1	1	0	0	1	3.23	<0.01-17.58
**Anhui**	22	0	0	0	0	0	0	-
**Jilin**	372	5	14	14	6	34	9.14	6.59-12.53
**Age (years)**								
**≤1**	50	0	1	0		1	2.00	<0.01-11.47
**>1**	375	6	14	14	6	34	9.07	6.53-12.43
**Total**	425	6	15	14	6	35	8.24	5.95-11.26
**Neospora caninum**								
**Region**								
**Henan**	31	0	0	0	0	0	0	**-**
**Anhui**	22	0	0	0	0	0	0	**-**
**Jilin**	43	0	2	3	1	6	13.95	6.18-27.64
**Age (years)**								
**≤1**	50	0	0	0	0	0	0	-
**>1**	46	0	2	3	1	6	13.04	5.74-26.04
**Total**	96	0	2	3	1	6	6.25	2.63-13.23

### Ethical aspects

This study was approved by the institutional animal use protocol committee of the United States Department of Agriculture and the Henan Agriculture University, China.

### Climatic conditions

The climate of Henan (Latitude 34.90°N, Longitude 113.50°E) is humid subtropical whereas the climate of Anhui province (Latitude 31.86°N, Longitude 117.28°E) is semi-humid, monsoonal, and the climate of Jilin province (Latitude 43.70°N, Longitude 126.20°E) is humid continental climate, winters are long (lasting from November to March), cold, and windy.

### Serologic examination

Serum samples from 425 dogs were tested for antibodies to *T. gondii* using the modified agglutination test (MAT) [5]. Serum samples from first batch samples (96 dogs) were also tested for *N. caninum* antibodies by the *Neospora* agglutination test (NAT) [6]. A titer of 1:25 was considered as indicative of exposure to both parasites. Sera were diluted with phosphate-buffered saline and tested 1:25, 1:50, 1:100 and 1:200 dilutions for both parasites. In addition, for *T. gondii*, sera were also tested at 1:10 dilution.

### Bioassay of tissues for *T. gondii*

Myocardium (50 g, or 30 g for young dog) from the first batch samples with MAT of *T. gondii* seropositive (MAT, ≥10) dogs (n = 14) was digested individually in pepsin and bioassayed in mice as described [7]. Briefly, tissues were washed and homogenized in saline (0.85% NaCl), mixed with acidic pepsin, and incubated in a shaker water bath for 60 min at 37°C. The homogenate was filtered through two layers of gauze, centrifuged, sediment neutralized with sodium bicarbonate, centrifuged again, mixed with antibiotics, and the homogenate inoculated subcutaneously into three Swiss Webster (SW) outbreed albino mice, and two gamma interferon gene knockout (KO) mice [7]. All inoculated mice were observed daily for illness. Dead mice, or killed when ill, were examined for *T. gondii* by making impression smears from the lung and examined for tachyzoites. Survivors were bled on day 41 post-inoculation (p.i.) and 1:100 dilution of serum from each mouse was tested for *T. gondii* antibodies with the MAT. Mice were killed 47 or 48 days post infection and brains of all mice were examined for tissue cysts as a squash preparation as described [7].

## Results and discussion

Six (6.25%) of 96 dogs were seropositive of *N. caninum* with titers of 1:25 in 2, 1:50 in 3, and 1:100 in 1, and all seropositive dogs were more than 1 year old (Table 1). Antibodies to *N. caninum* were found in 6 (13.95%) of 43 dogs from Jilin but not in 53 dogs from the Henan and Anhui, because most of the dogs from other two regions were less than one year old. Antibodies to *T. gondii* were found in 35 of 425 (8.24%) dogs with titers of 1:25 in 15, 1:50 in 14; and 1:100 in 6, none was positive at 1:200 (Table 1). The age range of the *N. caninum* and *T. gondii* negative dogs was 1.06 ± 0.55 (years) and 2.90 ± 1.38 (years). The age range of the *N. caninum* and *T. gondii* positive dogs was 2.25 ± 1.42 (years) and 3.06 ± 2.16 (years). T. gondii was not isolated in mice inoculated with tissues of any dogs.

Hearts samples and serum were kept cold and transported by air from China to USA within one week of killing. One week antibodies are fairly stable. *T. gondii* antibodies were stable on dried filter papers for up to 45 days at room temperature [8] and for 6 months at 25°C when stored with silica gel [9]. In an unpublished experiment antibodies were still detectable in blood 7 days after they were accidently left in a water bath at 37°C (JP Dubey, unpublished 1971).

In the present study, antibodies to *N. caninum* were detected in dog sera for the first time from China. How dogs become infected with *N. caninum* in nature is unknown [1]. Congenital transmission and ingestion of infected tissues or oocysts are the three probable modes of transmission. Of these, the ingestion of infected tissues is considered the most important because congenital transmission in dogs is relatively rare and fecal transmission has not been proven [3]. Little is known of the natural epidemiology of *N. caninum* in nature. In the USA, cattle and deer are commonly infected with *N. caninum*. Viable parasite has not been isolated from small mammals. Little is known of the epidemiology of neosporosis in China. In this study, antibodies to *N. caninum* were 6.25% (6/96) of first batch samples tested. We didn’t test the second batch samples because of non availability of reagents for testing of *N. caninum* by NAT, or IFA, or cELISA in China. Compared with other countries [10-13], the prevalence of *N. caninum* antibody was low in East Asia [14,15]; also it is low in this study. Although a comparison of different serological tests (NAT, IFA, cELISA) for the detection of *N. caninum* antibodies in dogs has not been made, it is generally accepted that results by NAT and IFA are comparable because both tests assess antibodies directed against whole tachyzoites [1]. None of the serological tests, however, have been validated because it is very difficult to isolate *N. caninum* from asymptomatic hosts [1].

**Figure 1:**
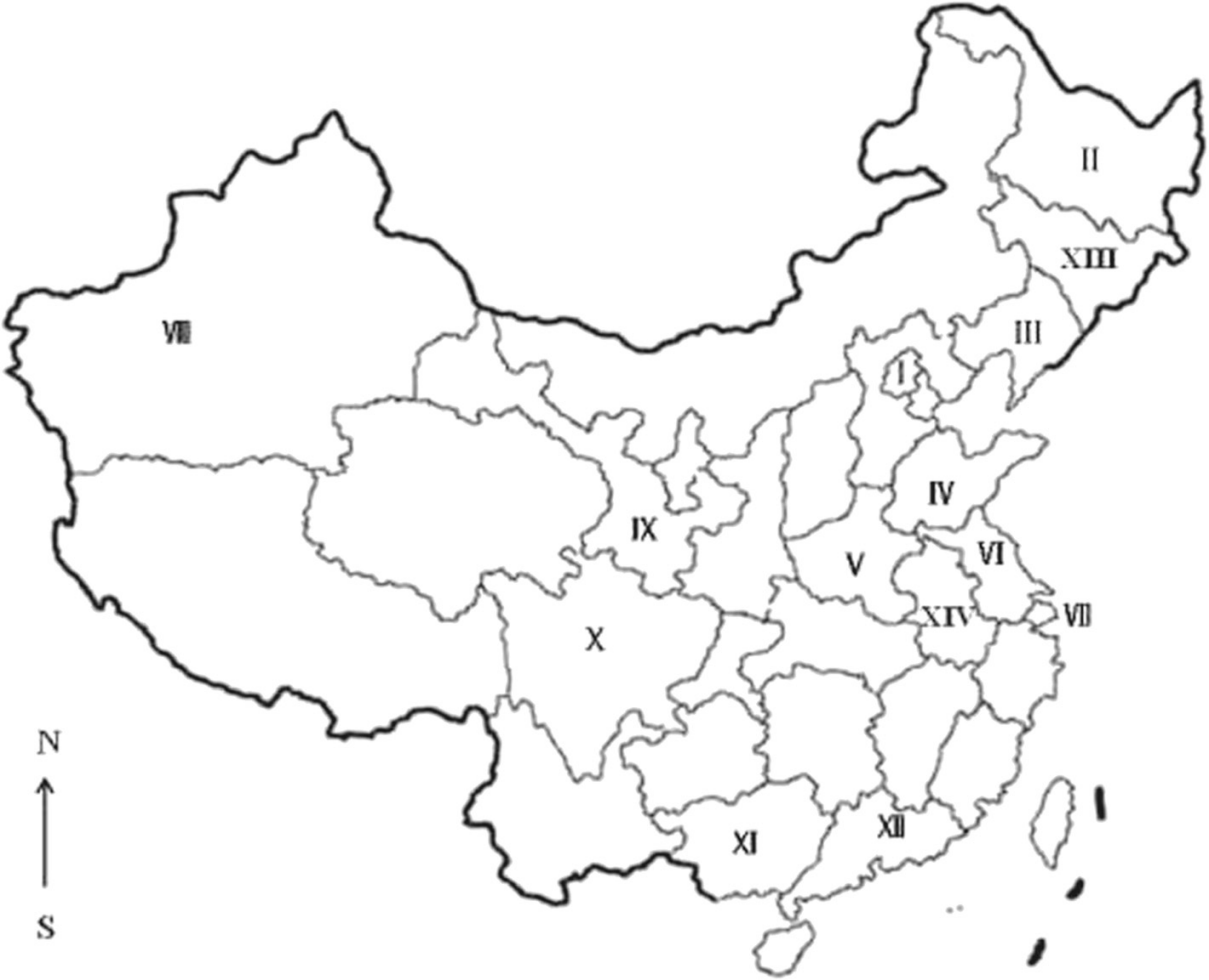
**Seroepidemiology of** ***Toxoplasma gondii*** **in dogs in China.** I: Beijing, II:Heilongjiang, III:Liaoning, IV:Shandong, V:Henan, VI: Jiangshu, VII:Shanghai, VIII:Xinjiang, IX:Gansu, X:Sichuan, XI:Yunnan, XII:Guangdong, XIII: Jilin, XIV: Anhui.

In this study, the prevalence of antibodies and titers to *T. gondii* were low compared to data from other parts of China (Table 2, Figure 1) and other country [16,17]. However, results are not strictly comparable because of different serological tests used and different cut-off used. In several of these previous reports, the serological tests used have not been critically evaluated. To facilitate further investigations we have summarized available reports on canine toxoplasmosis in Table 2. The MAT we used has been extensively used for the detection of *T. gondii* antibodies in many species, including humans and dogs [7]. Viable *T. gondii* was isolated from 51% (22/43) of dogs with MAT antibodies [18] and there is no evidence for any cross reactivity with other antigens in MAT [19]. The lack of isolation of *T. gondii* from the hearts of any of the dogs in the present study could be related to the loss of infectivity during transit from China and USA. However, viable *T. gondii* has been isolated in the USDA laboratory in Beltsville from tissues of animals that had been in transit for up to 10 days from several countries to USA. The survival of *T. gondii* tissue in meat at room temperature is largely unknown. However, viable *T. gondii* was isolated from a Hawaiian bird that was completely rotten and most carcasses had been eaten by maggots [20]. At 40°C, *T. gondii* tissue cysts survived up to 2 months [21]. Temperatures at which the dog tissues samples were stored varied from 4-20°C while in transit from China to USA. Therefore, we are uncertain of the effect of storage on the survival of tissue cysts.

**Table 2:** **Prevalence of** ***T. gondii*** **antibodies in dogs in People’s Republic of China**

**Map region**	**Provinces/cities**	**Year tested**	**Dog type**	**No. tested**	**No. positive**	**% positive**	**Serologic test**	**No. with terminal titer of**					**Reference**
								**<25**	**25 ~ 49**	**50 ~ 99**	**100 ~ 199**	**≥200**	
I	Beijing	1999-2005	Pet	534	128	24.0	ELISA^a^/LAT^b^	unknown					[22]
II	Heilongjiang	2010-2011	Stray/pet	124	14	11.3	IHA^b^	unknown					[23]
III	Liaoning	2012	Pet	328	33	10.0	IHA^b^	unknown					[24]
		2012	Police	291	90	30.9	MAT^e^	45	27	0	12	6	[25]
IV	Shangdong	2010-2011	Stray/pet	143	14	9.8	IHA^b^	unknown					[23]
V	Henan	2010-2011	Stray/pet	106	13	12.3	IHA^b^	unknown					[23]
		2013	Countryside	31	1	3.2	MAT^e^	1	1	0	0	0	Present study
VI	Jiangshu	unknown	Stray/countryside	231	93	40.3	ELISA^a^	unknown					[26]
		2010	Household	288	62	21.5	MAT^e^	21	15	0	11	9	[27]
VII	Shanghai	2009	City pet	1178	15	1.3	IHA^b^	unknown					[28]
			Town pet	364	22	6.0							
			Countryside	194	19	9.8							
VIII	Xinjiang	2010-2011	Stray/pet	259	29	11.2	IHA^b^	unknown					[23]
IX	Gansu	2010	Pet	259	28	10.8	MAT^e^	14	9	4	1	0	[29]
X	Sichuan	2010	Household	314	11	3.5	IHA^b^	0	0	4	3	4	[30]
XI	Yunnan	2011-2012	Pet	611	132	21.6	IHA^c^	0	0	29	25	78	[31]
XII	Guangdong	unknown	Stray	36	12	33.3	ELISA^d^	unknown					[32]
			Household	114	20	17.5							
XIII	Jilin	2013	Countryside	43	7	16.3	MAT^e^	5	6	1	0	0	Present study
		2014	Countryside	329	27	8.2		0	8	13	6	0	
XIV	Anhui	2013	Countryside	22	0	0	MAT^e^	0	0	0	0	0	Present study

IHA: indirect hemagglutination test, ELISA: enzyme-linked immunosorbent assay, LAT: latex agglutination test.

^a^:ELISA Kit produced by Zhuhai S.E.Z Haitai Pharmaceutlcals Co., Ltd, China.

^b^:Kit produced by Lanzhou Veterinary Institute, Academy of Agriculture and Science, China.

^c^:Kit produced by Nanjing Veterinary research Institute, Jiangshu Academy of Agriculture and Science, China.

^d^:Kit produced by Combined Company, Shenzhen, China.

^e^:in-house.

## Conclusions

Results of the present study indicated low prevalence of *N. caninum* and *T. gondii* infection in dogs in China, compared with data from Europe and America. Identification of the risk factors that underlie these differences may help prevention of neosporosis and toxoplasmosis. Dog meat is consumed by local people in China. Therefore, dogs are at a risk of *T. gondii* infection and should be of public health concern.
